# Activation and Proteolytic Activity of the *Treponema pallidum* Metalloprotease, Pallilysin

**DOI:** 10.1371/journal.ppat.1002822

**Published:** 2012-07-26

**Authors:** Simon Houston, Rebecca Hof, Lisa Honeyman, Julia Hassler, Caroline E. Cameron

**Affiliations:** Department of Biochemistry and Microbiology, University of Victoria, Victoria, British Columbia, Canada; Medical College of Wisconsin, United States of America

## Abstract

*Treponema pallidum* is a highly invasive pathogen that undergoes rapid dissemination to establish widespread infection. Previous investigations identified the *T. pallidum* adhesin, pallilysin, as an HEXXH-containing metalloprotease that undergoes autocatalytic cleavage and degrades laminin and fibrinogen. In the current study we characterized pallilysin's active site, activation requirements, cellular location, and fibrin clot degradation capacity through both *in vitro* assays and heterologous treponemal expression and degradation studies. Site-directed mutagenesis showed the pallilysin HEXXH motif comprises at least part of the active site, as introduction of three independent mutations (AEXXH [H^198^A], HAXXH [E^199^A], and HEXXA [H^202^A]) abolished pallilysin-mediated fibrinogenolysis but did not adversely affect host component binding. Attainment of full pallilysin proteolytic activity was dependent upon autocatalytic cleavage of an N-terminal pro-domain, a process which could not occur in the HEXXH mutants. Pallilysin was shown to possess a thrombin cleavage site within its N-terminal pro-domain, and *in vitro* studies confirmed cleavage of pallilysin with thrombin generates a truncated pallilysin fragment that has enhanced proteolytic activity, suggesting pallilysin can also exploit the host coagulation process to facilitate protease activation. Opsonophagocytosis assays performed with viable *T. pallidum* demonstrated pallilysin is a target of opsonic antibodies, consistent with a host component-interacting, surface-exposed cellular location. Wild-type pallilysin, but not the HEXXA mutant, degraded fibrin clots, and similarly heterologous expression of pallilysin in the non-invasive spirochete *Treponema phagedenis* facilitated fibrin clot degradation. Collectively these results identify pallilysin as a surface-exposed metalloprotease within *T. pallidum* that possesses an HEXXH active site motif and requires autocatalytic or host-mediated cleavage of a pro-domain to attain full host component-directed proteolytic activity. Furthermore, our finding that expression of pallilysin confers upon *T. phagedenis* the capacity to degrade fibrin clots suggests this capability may contribute to the dissemination potential of *T. pallidum*.

## Author Summary

Syphilis, caused by the spirochete *Treponema pallidum*, is a chronic sexually transmitted disease which infects 12 million people annually. *Treponema pallidum* is highly invasive and undergoes widespread dissemination via the circulatory system. Similar to other invasive pathogens, *T. pallidum* has been shown to express a host-component-degrading protease, pallilysin, that binds and degrades human fibrinogen and laminin, suggesting a role for pallilysin in bacterial dissemination. Here we identify pallilysin active site residues using mutagenesis and show that, unlike wild-type, mutants fail to degrade fibrinogen. We show that pallilysin is converted into a highly proteolytically active form via truncation of a pro-domain through either autocatalytic cleavage or host-derived, thrombin-mediated cleavage. We also demonstrate that recombinant pallilysin enables clot dissolution and that pallilysin expressed on the surface of the non-invasive spirochete *Treponema phagedenis* confers the ability to degrade fibrin clots. Further, we show that pallilysin is present on the surface of *T. pallidum* and thus resides in a cellular location that facilitates direct contact with host components. Our study provides insight into the mechanism of interaction between pallilysin and two important coagulation system proteins, fibrinogen and thrombin, and suggests a novel mechanism that *T. pallidum* may utilize for dissemination during infection.

## Introduction

The expression of host-interacting proteases has been shown to contribute to the pathogenesis of bacteria of medical interest by promoting host colonization and immune evasion, acquisition of nutrients, tissue invasion and dissemination of infection. Several pathogenic bacteria, including *Streptococcus pneumoniae* [1], *Yersinia pestis* [2], *Vibrio cholerae* [3], and *Clostridium perfringens* [4], express bacterial proteases and toxins which play a central role in the infection process through facilitation of bacterial dissemination and tissue invasion by proteolytic degradation of host proteins. Bacterial proteases and toxins have been shown to target a wide array of host molecules, including the extracellular matrix (ECM) components collagen [4], elastin [5], fibrinogen [6], fibronectin [7] and laminin [8]. Although host component degradation is common to many disseminating pathogens, such a mechanism has yet to be confirmed within the highly invasive causative agent of syphilis, *Treponema pallidum* subsp. *pallidum*.

Numerous studies have demonstrated that *T. pallidum* is capable of gaining rapid entry to the circulatory system following infection, with subsequent dissemination to distant host sites [9]–[14]. The highly invasive nature of the pathogen is further emphasized by the diverse clinical manifestations that can occur in untreated syphilis infections, including skin rashes, meningitis, ocular disease, and cardiovascular and neurological complications, and by the fact that *T. pallidum* can cause bone destruction in congenital and tertiary stage syphilis [15]. Furthermore, *T. pallidum* is one of only a few pathogens that can traverse the placental and blood-brain barriers.

Previously our laboratory identified the *T. pallidum* laminin-binding adhesin Tp0751 (GenBank accession number, AAC65720; also referred to as ‘pallilysin’) [16], [17]. Since laminin is an abundant glycoprotein component of the blood-brain barrier and basement membranes underlying endothelial cell layers, barriers which *T. pallidum* must traverse during the course of infection, pallilysin was proposed to contribute to the *T. pallidum* infection process [16]. Pallilysin-specific antibodies have been detected in serum from both natural and experimental *T. pallidum* infections [16], indicating that the protein is expressed during the course of infection. Additionally, heterologous expression of pallilysin on the surface of the culturable non-adherent spirochete, *Treponema phagedenis*, conferred upon the bacterium the ability to bind laminin, a specific interaction which was inhibited by the presence of pallilysin-specific antibodies [18].

Recently we have shown that pallilysin is also capable of specific binding to human fibrinogen [19], a key structural protein in blood coagulation. A second central component of the coagulation cascade is thrombin, an abundant serine protease that catalyzes the conversion of soluble fibrinogen to insoluble fibrin, the major structural component of haemostatic clots and abscesses [20]. Expression of both fibrinogen and thrombin is significantly up-regulated as a host response to inflammation and infection, and the coagulation process is critical for pathogen containment during infection [21]. Several pathogenic bacteria have developed strategies to overcome this host defence mechanism, including expression and activation of fibrinogenolytic proteases [22]–[24]. Previously we showed that pallilysin degrades human fibrinogen and laminin, and consistent with this finding we identified a putative metalloprotease motif (HEXXH) within the C-terminus of pallilysin [19].

Prior investigations also indicated pallilysin is capable of being activated via autocatalysis [19], a common activation mechanism for ECM-degrading matrix metalloproteases (MMPs) [25]. Bacterial metalloproteases whose activity is regulated by autocatalysis, such as thermolysin-like metalloproteases [26], [27], are initially synthesized as an inactive pre-pro-protease consisting of a signal (pre-) sequence, pro-domain, and mature active protease domain(s). The major role of the pro-domain, which is cleaved to form the active enzyme, is to mediate correct protein folding and to maintain the protease in an inactive form to prevent premature activation prior to attainment of its target location [28]. Numerous eukaryotic pro-proteases, such as the pro-matrix metalloproteases (pro-MMPs)-2 and -9, are cleaved into their active mature form by the action of another protease, including other MMPs [29]. To our knowledge, there have not been any prior reports of inter-molecular activation of a bacterial metalloprotease via a host protease-mediated processing event.

In the current study we elucidate pallilysin's mechanisms of activation and proteolysis via identification of key active site residues using a site-directed mutagenesis-based approach. We demonstrate that, in contrast to wild-type pallilysin, alanine-substituted HEXXH mutant forms of the protease fail to degrade fibrinogen and insoluble fibrin clots, indicating that the C-terminal HEXXH motif comprises, at least in part, the metalloprotease active site. We identify contiguous thrombin and autocatalytic cleavage sites in the N-terminus of pallilysin and demonstrate that mature active pallilysin is formed by removal of an N-terminal pro-domain via either inter-molecular autocatalytic cleavage or thrombin cleavage. Furthermore, we show that heterologous expression of pallilysin on the surface of *T. phagedenis* confers upon the transformed bacteria the capacity to degrade insoluble fibrin clots. Finally, we demonstrate that pallilysin is a target of opsonic antibodies, suggesting that the protease is also surface-exposed in *T. pallidum* and thus capable of directly interacting with host components during infection.

## Results

### Zinc addition during pallilysin purification alters the pallilysin SDS-PAGE migration profile

**Figure 1:**
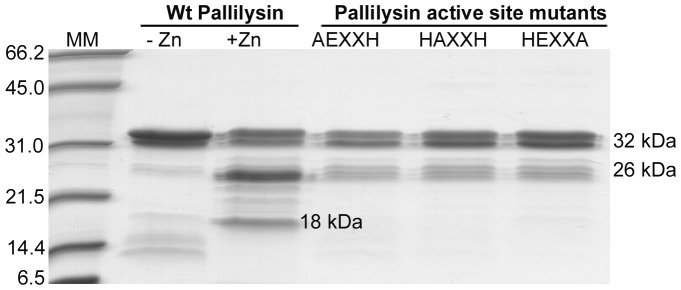
Purification with zinc affects the migration of recombinant pallilysin. SDS-PAGE analysis of purified recombinant wild-type His-tagged pallilysin (C^24^-P^237^) purified in the absence (-Zn) and presence (+Zn) of zinc and pallilysin (C^24^-P^237^) active site mutants (AEXXH [H^198^A], HAXXH [E^199^A], HEXXA [H^202^A]) purified in the presence of Zn. Each lane contains approximately 6 µg of protein. Numbers to the left of the lanes indicate the size (kDa) of the corresponding molecular mass (MM) markers.

In order to investigate the potential activation and proteolytic mechanisms of pallilysin, several soluble recombinant proteins were expressed and purified in *E. coli*; (1) wild-type full-length (C^24^-P^237^) pallilysin purified in the presence and absence of zinc and calcium and (2) three full-length (C^24^-P^237^) pallilysin active site mutants (AEXXH [H^198^A], HAXXH [E^199^A], and HEXXA [H^202^A]) purified in the presence of zinc and calcium. In agreement with previous findings [19], and as shown in **Figure 1**, wild-type pallilysin migrated as multiple bands via SDS-PAGE analysis. Major bands were consistently detected at approximately 32 kDa, 26 kDa, and 18 kDa (wild-type pallilysin in presence of zinc and calcium only), with the 26 kDa protein band being the dominant wild-type pallilysin form detected following purification in the presence of zinc and calcium. In contrast, the 32 kDa protein band was the dominant form detected in the pallilysin mutants (**Figure 1**). Mass spectrometry analyses (data not shown) and Edman sequencing, as described below, identified all bands as pallilysin, indicating that all recombinant proteins were purified to homogeneity. The major band corresponding to 32 kDa was identified as full-length pallilysin and the 26 kDa and 18 kDa peptides as truncated forms of pallilysin.

### Residue H^198^, but not E^199^, from the pallilysin HEXXH motif is required for zinc coordination

To determine whether the previously identified pallilysin C-terminal HEXXH motif [19] is required for zinc coordination, one of the potential pallilysin zinc-coordinating mutants (AEXXH [H^198^A]) and the potential pallilysin catalytic general acid/base mutant (HAXXH [E^199^A]) were analyzed for their ability to bind divalent zinc. This was accomplished via quantitative inductively coupled plasma-mass spectrometry (ICP-MS), a highly sensitive mass spectrometry technique that determines the concentration of trace metals in a given sample at concentrations as low as one part per trillion. As expected, these analyses indicate wild-type pallilysin and the HAXXH (E^199^A) mutant, but not the AEXXH (H^198^A) mutant, bind zinc (**Table 1**). For HEXXH-containing proteins that bind zinc, stoichiometries of approximately 1.0 would be expected. In the current study, the lower than expected stoichiometries were likely due to the fact that zinc could not be added during pallilysin expression as this resulted in high levels of pallilysin adherence to gel filtration columns and very low pallilysin yields. Furthermore, non-His-tagged pallilysin binds to nickel affinity columns (data not shown), presumably through the HEXXH motif, suggesting nickel may competitively inhibit coordination of zinc to the pallilysin HEXXH motif. In the current study, lower zinc levels were detected in wild-type pallilysin compared to previous findings [19]. This is likely due to the variable levels of zinc available for protease coordination during protein expression, primarily resulting from variable zinc concentrations in the bacterial growth media.

**Table 1:** ICP-MS quantification of zinc content in wild-type and mutant pallilysin.

Protein	Zinc concentration (nM)	Protein concentration (nM)	Ratio of [metal ion]∶[protein]
Pallilysin C^24^-P^237^ HEVIH (wt)	4.4	30.0	0.15∶1.0
Pallilysin C^24^-P^237^ AEVIH	ND	30.0	NA
Pallilysin C^24^-P^237^ HAVIH	6.3	30.0	0.21∶1.0

ND: Not detected.

NA: Not applicable.

Detection limit: 1.0 part per billion (ppb).

### Pallilysin binds fibrinogen and laminin in a dose-dependent manner

**Figure 2:**
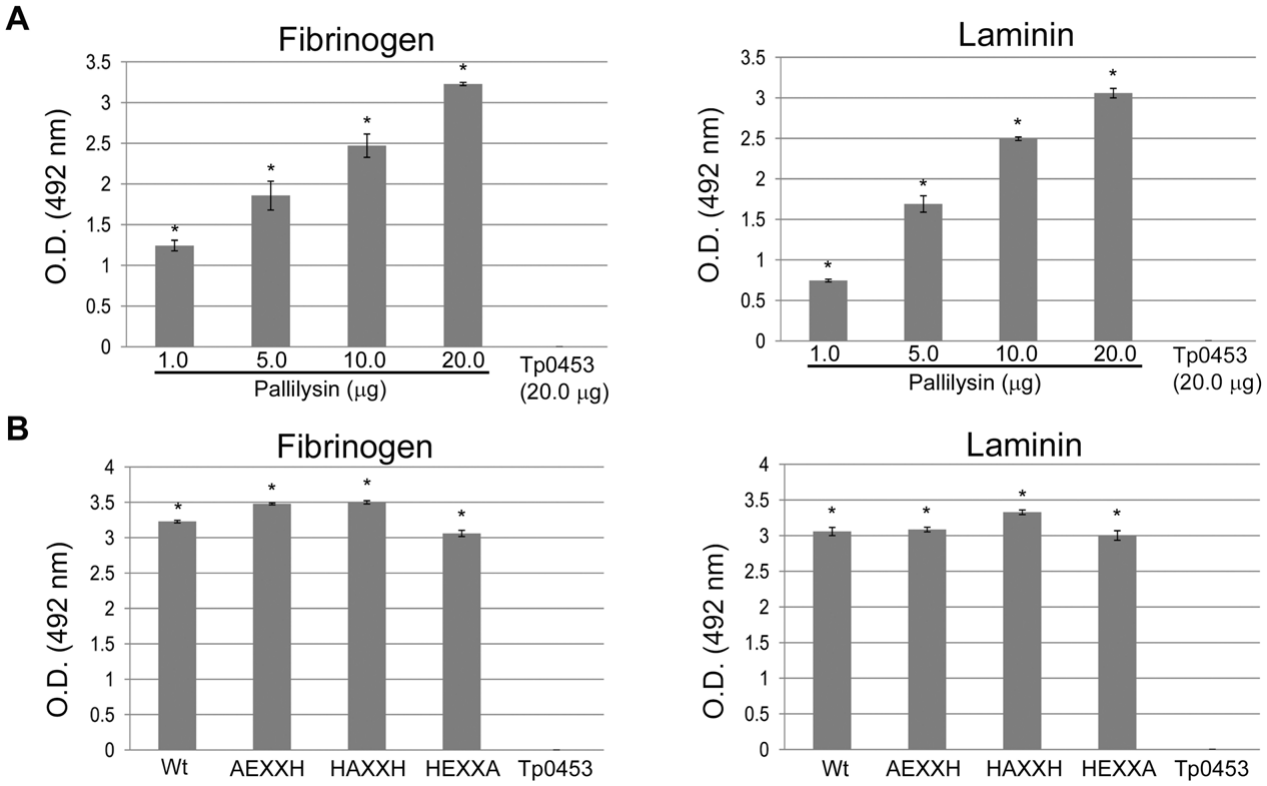
Fibrinogen- and laminin-binding by pallilysin is specific and is not adversely affected by HEXXH mutations. (A) An ELISA-based assay was used to assess the effect of varying concentrations of wild-type pallilysin (0–20 µg per well) on binding to fibrinogen (0.5 µg per well) (left panel) or laminin (0.5 µg per well) (right panel). Recombinant Tp0453 (20 µg) was used as the negative control. (B) The ability of each pallilysin HEXXH mutant, wild-type pallilysin, and the negative control Tp0453 (20 µg each per well) to attach to immobilized human fibrinogen (left panel) or laminin (0.5 µg each per well) (right panel) was assessed using an ELISA-based assay. Average readings from triplicate measurements are presented with bars indicating standard error (SE). The results are representative of two independent experiments. For statistical analyses, attachment to laminin and fibrinogen by wild-type and mutant pallilysin was compared to the attachment of Tp0453 using the Student's two-tailed *t* test. The three pallilysin HEXXH mutants and all concentrations of wild-type pallilysin (0–20 µg) exhibited statistically significant levels of binding to laminin and fibrinogen compared to the level of binding exhibited by Tp0453 (* indicates *p*<0.0001).

An ELISA-based approach was used to assess the binding specificity of soluble wild-type pallilysin (C^24^-P^237^) to immobilized fibrinogen and laminin as a function of varying pallilysin concentrations (1–20 µg). As shown in **Figure 2A**, increasing concentrations of pallilysin bound to both fibrinogen and laminin in a dose-dependent manner. Furthermore, all concentrations of wild-type pallilysin (1–20 µg) exhibited statistically significant levels of binding to both fibrinogen and laminin (*p*<0.0001) when compared to the level of binding exhibited by the negative control, Tp0453 [30] (20 µg) (GenBank accession number; AAC65443).

### Pallilysin HEXXH active site mutants bind human fibrinogen and laminin

The ability of the three pallilysin HEXXH active site mutants to exhibit attachment to fibrinogen and laminin was investigated using the ELISA-based assay. As shown in **Figure 2B**, wild-type pallilysin and all three pallilysin HEXXH mutants exhibited similar, statistically significant levels of binding to fibrinogen and laminin (*p*<0.0001) when compared to the level of binding exhibited by Tp0453. These results indicate that binding of pallilysin to fibrinogen and laminin is HEXXH-independent, and therefore cannot be solely attributed to a transient metalloprotease-substrate interaction.

### Pallilysin HEXXH active site mutants fail to degrade fibrinogen

**Figure 3:**
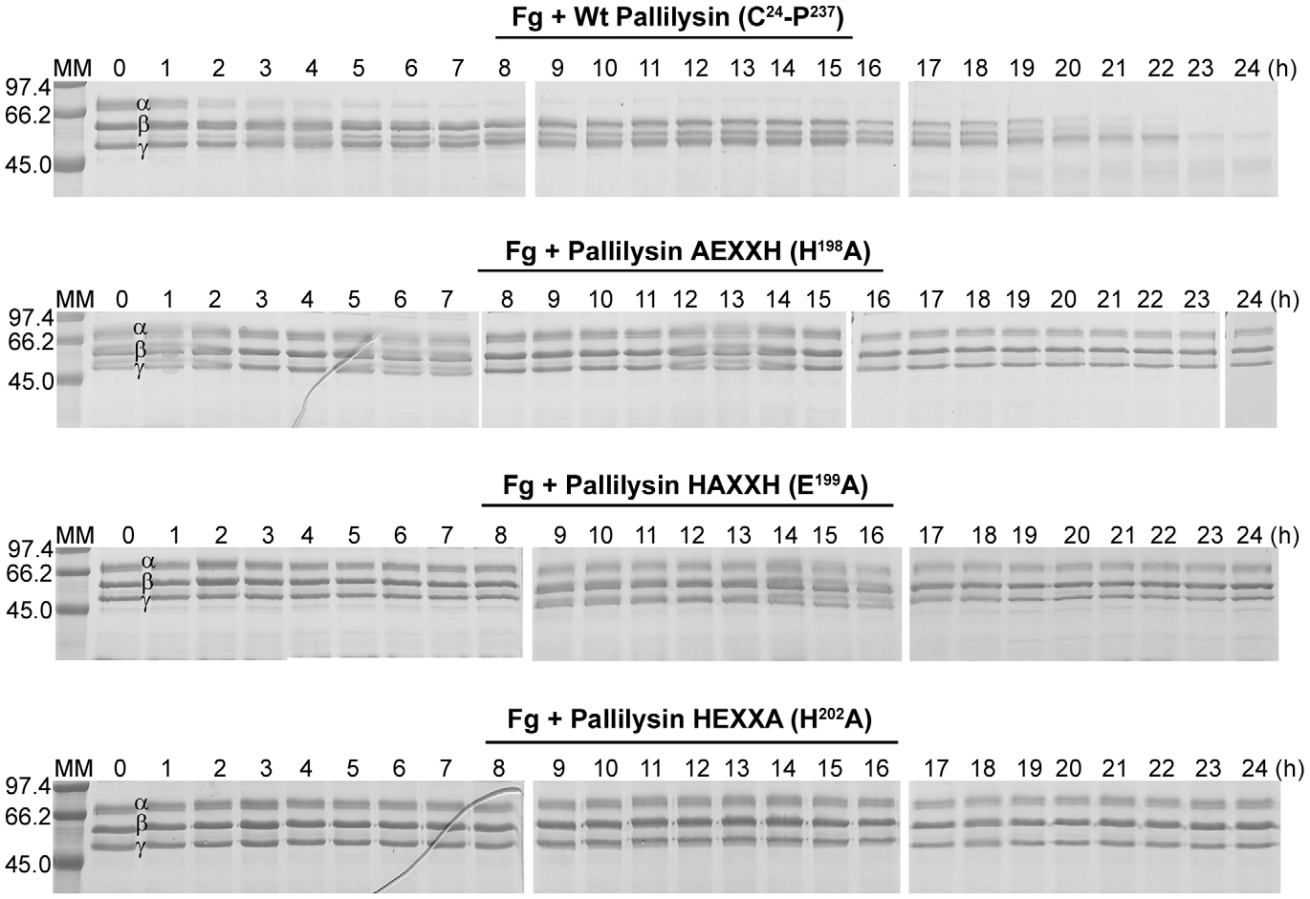
Site-directed mutagenesis of the pallilysin HEXXH histidine and glutamic acid residues abolishes fibrinogenolytic activity. Recombinant wild-type pallilysin (C^24^-P^237^) and the three pallilysin mutants (AEXXH [H^198^A], HAXXH [E^199^A], and HEXXA [H^202^A]) (300 µg) were incubated with plasminogen-free human fibrinogen (600 µg) at 37°C for 24 h. Samples were removed every hour from 0–24 h post-incubation and analyzed by SDS-PAGE for degradation of the fibrinogen α-, β-, and γ-chains. Negative controls included fibrinogen incubated alone (Fg only) and fibrinogen incubated in the presence of Tp0453 (data not shown). Each lane contains approximately 6 µg of protein. Numbers to the left of the lanes indicate the size (kDa) of the corresponding molecular mass (MM) markers.

To determine the effect of the introduced HEXXH mutations on pallilysin-mediated host protein degradation, wild-type pallilysin and the three HEXXH mutants were incubated with fibrinogen for 24 h at 37°C. Samples were removed at hourly intervals and the fibrinogenolytic activity of each protein was determined by gel-based analyses. Substitution of each of the three predicted active site residues with alanine abolished the fibrinogenolytic activity of the corresponding mutants (**Figure 3**). Wild-type pallilysin initiated fibrinogen degradation within 1 h of incubation, with complete degradation of the fibrinogen α-, β-, and γ-chains observed by 23 h post-incubation, whereas the corresponding mutants failed to degrade any of the three fibrinogen chains over the 24 h duration of the experiment (**Figure 3**). The negative control, Tp0453, did not exhibit fibrinogen degradation (data not shown). These results indicate that the pallilysin C-terminal HEXXH motif resides within the protease active site.

### Wild-type pallilysin undergoes autocatalytic cleavage

**Figure 4:**
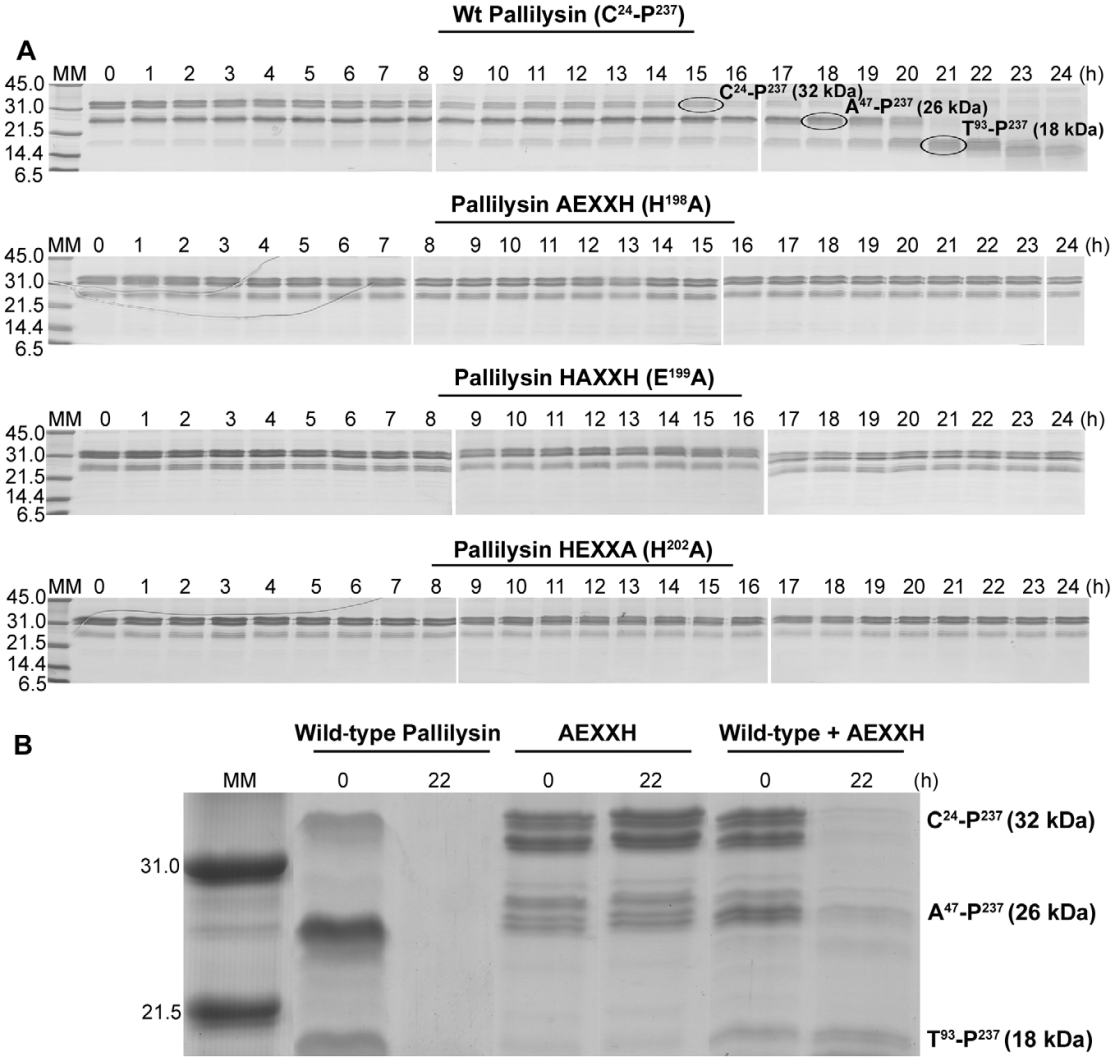
Pallilysin is autocatalytically activated via sequential inter-molecular cleavages at residues T^46^ and Q^92^. (A) Recombinant wild-type pallilysin (C^24^-P^237^) and active site mutant pallilysin (AEXXH [H^198^A], HAXXH [E^199^A], and HEXXA [H^202^A]) purified from *E. coli* (500 µg) were incubated at 37°C for 24 h. Samples (10 µg) were removed every hour from 0–24 h post-incubation and analyzed by SDS-PAGE. The generated autocatalytic peptide bands corresponding to proteins ranging in mass from approximately 32 kDa to 18 kDa (circled) were analysed by Edman sequencing. The negative control Tp0453 failed to undergo autolysis (data not shown). (B) Purified recombinant wild-type (100 µg) and mutant (AEXXH [H^198^A]) pallilysin (100 µg) were incubated independently and together at 37°C for 22 h. Samples were removed at 0 and 22 h post-incubation and analyzed by SDS-PAGE for the presence or absence of autocatalytic peptides. Each lane contains approximately 6 µg of protein (in the absence of degradation). Numbers to the left of the lanes indicate the size (kDa) of the corresponding molecular mass (MM) markers.

As previously reported [19], SDS-PAGE analysis of purified recombinant pallilysin consistently results in the detection of multiple protein bands suggesting that pallilysin undergoes autocatalytic cleavage. Since we have demonstrated the HEXXH motif constitutes, at least in part, the metalloprotease active site, we predicted that substitution of the potential zinc binding histidines and catalytic glutamate with alanine would prevent autocatalytic cleavage of the mutant proteins. In order to test this prediction, wild-type and the three pallilysin HEXXH mutants were incubated independently for 24 h at 37°C, samples were removed at hourly intervals, and autocatalytic activity was assessed via SDS-PAGE analysis. Compared to wild-type pallilysin, the ability to undergo autocatalytic cleavage was abolished in each of the three pallilysin HEXXH mutants (**Figure 4A**). Incubation of wild-type pallilysin resulted in complete degradation of the major pallilysin bands (32 kDa and 26 kDa) by 21–22 h post-incubation with a concurrent increase in the intensity of the 18 kDa protein band (**Figure 4A**). Interestingly this timing correlates with maximal fibrinogen degradation (**Figure 3**). Conversely, the 32 kDa and 26 kDa protein bands from each of the three pallilysin HEXXH mutants remained stable throughout the course of the 24 h incubation (**Figure 4A**).

### Autocatalytic cleavage of pallilysin occurs between T^46^-A^47^ and Q^92^-T^93^

In order to identify the pallilysin autocatalytic cleavage sites, the 32, 26, and 18 kDa wild-type pallilysin protein bands generated during the 24 h incubation (**Figure 4A**) were analysed by Edman sequencing. As indicated in **Table 2**, the first 10 N-terminal amino acids identified from the 32 kDa protein band corresponded to the histidine tag originating from the expression vector, indicating that this represents full-length pallilysin (C^24^-P^237^). The 26 and 18 kDa protein bands matched the pallilysin amino acid sequence that would be expected following cleavage between residues T^46^-A^47^ and Q^92^-T^93^, respectively (**Table 2**). *In silico* analyses predict cleavage of this presumed “pro-domain” between T^46^-A^47^ and Q^92^-T^93^ generates peptides with approximate molecular masses of 21 kDa (A^47^-P^237^) and 16 kDa (T^93^-P^237^). However, all three pallilysin forms migrate on SDS-PAGE with slower-than-predicted mobility, a result consistent with previous findings demonstrating that pallilysin exhibits anomalous SDS-PAGE migration [19].

**Table 2:** Pallilysin N-terminal amino acids identified by Edman sequencing.

Peptide	Molecular mass on SDS-PAGE (kDa)	N-terminal amino acids identified by Edman sequencing
1 (full-length)	32	Ser – Tyr – Tyr – His – His – His – His – His – His - Leu
2 (A^47^-P^237^)	26	Ala – Ala – Leu – Pro – Ser – Asn – Ala – Arg – Asp – Thr
3 (T^93^-P^237^)	18	Thr – His – Thr – Gln – Pro – Pro – Val – Gln – Thr - Ala

### Pallilysin is capable of inter-molecular autocatalytic cleavage

To test whether pallilysin undergoes intra- and/or inter-molecular autocatalysis, mature active wild-type pallilysin was incubated with an equal concentration of the AEXXH-mutated protease for 22 h at 37°C. As shown in **Figure 4B**, wild-type pallilysin was capable of cleaving mutant pallilysin resulting in complete degradation by 22 h post-incubation. Mutant pallilysin (AEXXH) alone did not undergo autocatalytic cleavage, whereas complete autocatalytic proteolysis of wild-type pallilysin was observed following 22 h incubation at 37°C (**Figure 4B**). Degradation of the wild-type pallilysin 18 kDa protein band following 22 h incubation (**Figure 4B**, **lane 3**) is likely due to enhanced autocatalytic activity arising from the variable zinc concentrations that are present in the bacterial growth medium used during protein purifications. These results demonstrate that *in vitro* pallilysin autocatalysis occurs via two sequential cleavage events whereby residues C^24^–T^46^ are initially removed via inter-molecular cleavage, followed by a second inter-molecular cleavage event which removes residues A^47^–Q^92^.

### Pallilysin is activated via inter-molecular autocatalysis

**Figure 5:**
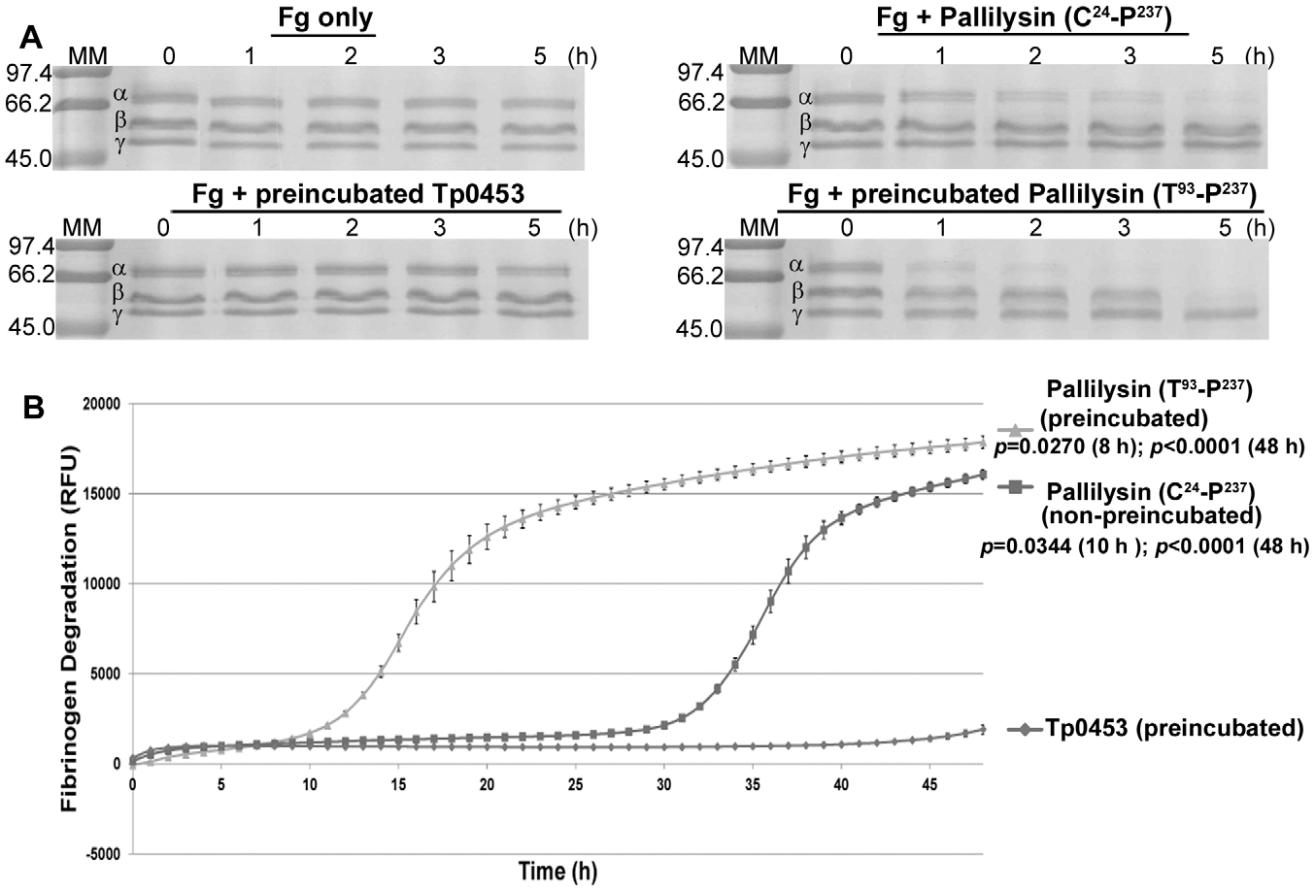
Autocatalysis enhances pallilysin-mediated fibrinogenolysis. (A) Wild-type pallilysin subjected to 22 h pre-incubation at 37°C (T^93^-P^237^) in the absence of substrate (30 µg) and non-pre-incubated wild-type pallilysin (C^24^-P^237^) (30 µg) were incubated with fibrinogen (60 µg) at 37°C for 5 h. Samples were removed at the times indicated and analyzed by SDS-PAGE to detect degradation of the fibrinogen α-, β-, and γ-chains. Negative controls included fibrinogen incubated in buffer alone (Fg only) and fibrinogen incubated in the presence of Tp0453, which had also been pre-incubated at 37°C for 22 h. Each lane contains approximately 6 µg of fibrinogen in the absence of proteolysis. Numbers to the left of the lanes indicate the size (kDa) of the corresponding molecular mass (MM) markers. (B) FITC-labelled fibrinogen (10 µg) was incubated at 37°C for 48 h in the dark with either non-pre-incubated wild-type pallilysin (C^24^-P^237^), pallilysin subjected to 22 h pre-incubation at 37°C (T^93^-P^237^) (1 µg), or the negative control Tp0453 (pre-incubated at 37°C for 22 h) (1 µg). The degree of fibrinogen degradation was measured every hour by detecting the increase in Relative Fluorescence Units (RFU) over 48 h using standard fluorescein excitation/emission filters. Average fluorescence intensity readings from octuplicate measurements are presented with bars indicating standard error (SE), and the results are representative of three independent experiments. For statistical analyses, fibrinogen degradation by wild-type pallilysin (non-pre-incubated [C^24^-P^237^] and pre-incubated [T^93^-P^237^]) was compared to that of Tp0453 (pre-incubated) using the Student's two-tailed *t* test. Pre-incubated (T^93^-P^237^) and non-pre-incubated (C^24^-P^237^) wild-type pallilysin exhibited a statistically significant level of fibrinogenolysis at 8 h (*p* = 0.0270) and 10 h (*p* = 0.0344) post-incubation, respectively, and beyond, compared to the level of fibrinogen degradation exhibited by the negative control, Tp0453.

To determine if wild-type pallilysin is in a more proteolytically active form following incubation and autocatalytic cleavage, wild-type pallilysin was pre-incubated at 37°C for 21 h, which generated the final autocatalytically cleaved mature protease (T^93^-P^237^). This version of the protease was incubated with fibrinogen for 5 h at 37°C, samples were removed at hourly intervals and the fibrinogenolytic activity was compared to that of a similarly pre-incubated negative control (Tp0453) and wild-type pallilysin (C^24^-P^237^) that had not undergone the pre-incubation step. As shown in **Figure 5A**, pre-incubated pallilysin (T^93^-P^237^) exhibited enhanced proteolytic activity over its non-pre-incubated (C^24^-P^237^) counterpart, as evidenced by accelerated fibrinogen α-chain degradation and complete β-chain degradation by 5 h post-incubation, a process which normally takes up to 20 h in the absence of a pre-incubation step for pallilysin (**Figure 3**). Pre-incubated Tp0453 failed to degrade fibrinogen (**Figure 5A**). Our previously established FRET-based fibrinogen degradation assay [19] was also performed to further compare the fibrinogenolytic activity of pre-incubated (T^93^-P^237^) and non-pre-incubated (C^24^-P^237^) pallilysin. As shown in **Figure 5B**, pre-incubated pallilysin (T^93^-P^237^) exhibited a statistically significant level of fibrinogenolysis at 8 h post-incubation (*p* = 0.0270) and beyond (*p*<0.0001 at 48 h), compared to the level of fibrinogen degradation exhibited by the negative control Tp0453. Non-pre-incubated pallilysin (C^24^-P^237^) exhibited a statistically significant level of fibrinogenolysis at 10 h post-incubation (*p* = 0.0344) and beyond (*p*<0.0001 at 48 h), compared to the level of fibrinogen degradation exhibited by the negative control. From this degradation curve it was evident that the majority of pre-incubated (T^93^-P^237^) pallilysin-mediated fibrinogenolysis occurred between 10–20 h post-incubation, whereas most non-pre-incubated (C^24^-P^237^) pallilysin-mediated fibrinogenolysis occurred approximately 20 h later during the 30–40 h post-incubation period. These results demonstrate that cleavage of pallilysin at Q^92^-T^93^ results in more rapid pallilysin-mediated degradation of fibrinogen.

### The N-terminus of pallilysin contains a thrombin cleavage site

**Figure 6:**
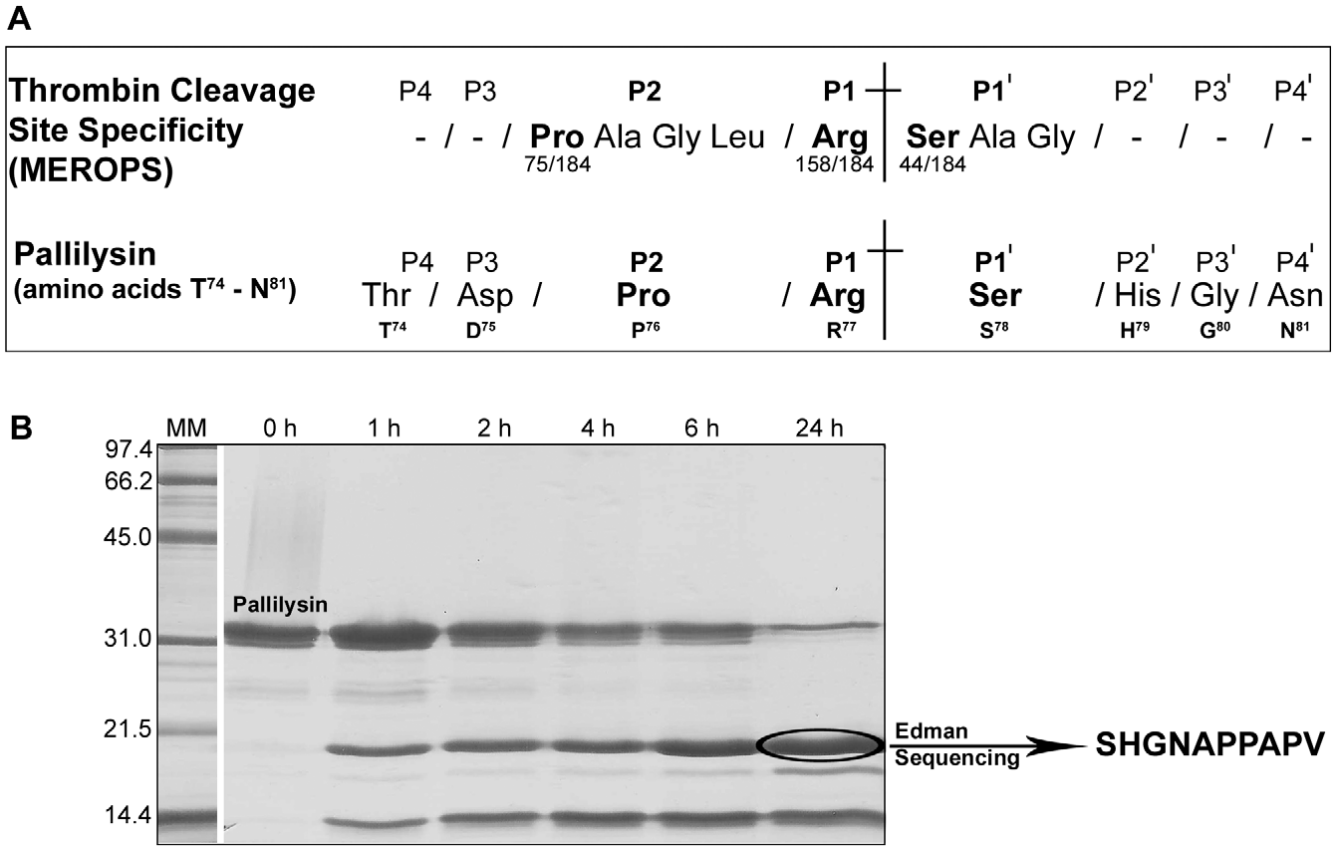
Pallilysin N-terminus is comprised of contiguous thrombin and autocatalytic cleavage sites. (A) The primary structure of pallilysin was manually analyzed for the presence of potential thrombin cleavage sites by comparison to the MEROPS thrombin cleavage site specificity matrix. Shown is a schematic representation of the only potential thrombin cleavage site detected within pallilysin, with substrate subsite residues P4–P4′ (T^74^–N^81^) indicated. Highlighted in bold are the most commonly found amino acid residues located at the thrombin substrate subsites P2, P1, and P1′ (based on experimental data from 184 thrombin cleavages [32]) which are compared to potential substrate subsites of pallilysin. (B) Purified recombinant wild-type pallilysin (C^24^-P^237^) (100 µg) was incubated with human thrombin (4.5 µg) at 37°C for 24 h. Samples (10 µg) were removed at the indicated intervals and analyzed by SDS-PAGE. The first 10 amino acids of the cleaved pallilysin protein band corresponding to a molecular mass of approximately 18 kDa (circled) were determined by Edman sequencing. The negative control, Tp0453, was not cleaved by thrombin during the same incubation period (data not shown). Numbers to the left of the lanes indicate the size (kDa) of the corresponding molecular mass (MM) markers.

Analysis of the pallilysin amino acid sequence for the presence of predicted protease cleavage sites using the ‘peptide cutter program’ [31] failed to identify any single potential cleavage site within pallilysin that could be targeted by commonly known proteases (data not shown). However, manual analysis of the pallilysin amino acid sequence by comparison to the thrombin cleavage site specificity matrix from MEROPS [32], a database which indicates the most common amino acid residues to be located in protease cleavage subsites, identified a predicted thrombin cleavage site between pallilysin residues R^77^ and S^78^ (**Figure 6A**). According to the MEROPS analysis, which at the time of writing was based on 184 thrombin cleavage reactions, the most common residues located in thrombin substrate subsites P2, P1, and P1′ are proline, arginine, and serine, respectively. All three residues were identified within the N-terminus of pallilysin (P^76^, R^77^, S^78^).

In order to experimentally confirm the predicted pallilysin thrombin cleavage site, full-length mature pallilysin (C^24^-P^237^) and the negative control, Tp0453, were incubated with thrombin at 20°C for 24 h. Protein samples were removed at 0, 1, 2, 4, 6, and 24 h post-incubation and analyzed for thrombin cleavage using SDS-PAGE. Thrombin cleaved mature recombinant pallilysin into two major peptides with molecular masses of approximately 18 kDa and 14 kDa (**Figure 6B**). Thrombin cleavage of the recombinant control, Tp0453, was not observed (data not shown). Given the fact that cleavage of pallilysin at the predicted thrombin cleavage site (R^77^-S^78^) would result in the generation of an approximately 18 kDa peptide, the protein band corresponding to this size was subjected to N-terminal amino acid sequence analysis using Edman degradation. As indicated in **Figure 6B**, the first 10 N-terminal amino acids identified matched the pallilysin sequence expected following thrombin cleavage at the R^77^-S^78^ site (SHGNAPPAPV). Substitution of the highly conserved arginine from the pallilysin thrombin cleavage P1 subsite with glycine (R^77^G) abolished the ability of thrombin to cleave pallilysin at the mutated internal thrombin site (data not shown). These results confirm the presence of an N-terminal thrombin cleavage site within pallilysin at residues R^77^-S^78^, a location which is just upstream of the final autocatalytic cleavage site (Q^92^-T^93^).

### Pallilysin truncated at the internal thrombin site retains fibrinogenolytic activity

**Figure 7:**
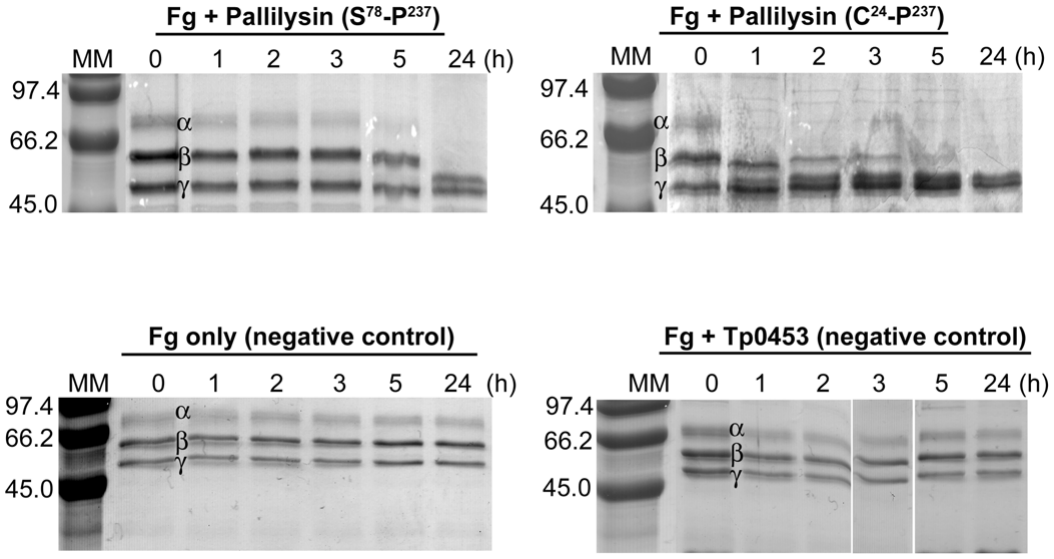
Pallilysin thrombin site truncation results in enhancement of fibrinogenolysis. Tagless pallilysin (S^78^-P^237^ and C^24^-P^237^) purified from *E. coli* (30 µg) was incubated with fibrinogen (60 µg) at 37°C for 24 h. Samples were analyzed by SDS-PAGE to detect degradation of the fibrinogen α-, β-, and γ-chains. Controls included fibrinogen (60 µg) incubated in buffer alone (Fg only) and fibrinogen (60 µg) incubated in the presence of the recombinant negative control Tp0453 (Fg + Tp0453) (30 µg). Each lane contains approximately 5 µg of protein (in the absence of fibrinogen degradation). Numbers to the left of the lanes indicate the size (kDa) of the corresponding molecular mass (MM) markers.

To test if the thrombin cleavage site within the N-terminus of pallilysin could also be involved in pallilysin activation, recombinant non-tagged pallilysin comprising residues C^24^-P^237^ (full-length pallilysin, includes N-terminal pro-domain) and S^78^-P^237^ (lacking N-terminal pro-domain due to truncation at internal thrombin cleavage site) were produced and their rate of fibrinogenolysis was compared using the SDS-PAGE-based fibrinogen degradation assay. As shown in **Figure 7**, truncation of pallilysin at the internal thrombin site did not adversely affect pallilysin proteolytic activity, in that the S^78^-P^237^ recombinant protein retained the capability to degrade the fibrinogen α- and β-chains. The negative controls comprising fibrinogen incubated in the absence of recombinant protein and in the presence of recombinant Tp0453 remained stable throughout the duration of the incubation period (**Figure 7**). Of particular interest, non-His-tagged full-length pallilysin (C^24^-P^237^) exhibited enhanced fibrinogenolysis, with complete α- and partial β-chain degradation by 5 h. This result is consistent with the concept that pro-domains are required to mediate correct protein folding [28] and our previous finding that removal of the N-terminal His-tag resulted in enhanced pallilysin-mediated fibrinogenolysis [19].

### Recombinant pallilysin and *T. phagedenis* heterologously expressing pallilysin degrade insoluble fibrin clots

**Figure 8:**
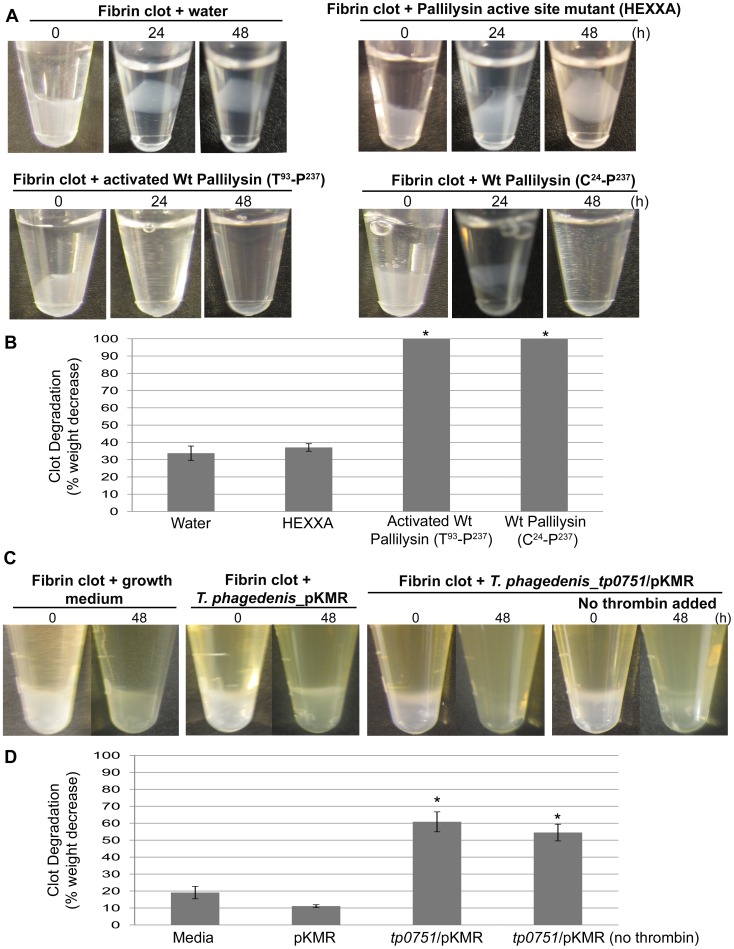
Pallilysin degrades fibrin clots. (A) Fibrin clots (generated from 300 µg fibrinogen) were incubated with water (negative control), pallilysin HEXXA (H^202^A) active site mutant (40 µg), autocatalytically activated wild-type pallilysin (T^93^-P^237^) (40 µg) or wild-type full-length pallilysin (C^24^-P^237^) (40 µg) for 48 h at 37°C in pallilysin activation buffer. After 0, 24, and 48 h, fibrin clot stability was analyzed visually. Results are representative of seven independent experiments. (B) Fibrin clot degradation by recombinant pallilysin was quantitated following 48 h incubation at 37°C. Clot weights were measured at 0 and 48 h post-incubation and percent clot degradation was determined. Mean readings from triplicate experiments are presented with bars indicating standard error (SE). Student's two-tailed *t* test indicated both forms of wild-type pallilysin exhibited statistically significant levels of fibrinolysis compared to the levels exhibited by water and HEXXA (* indicates *p*<0.0001). (C) Fibrin clots were incubated with TYGVS growth medium in the presence of thrombin (2 µg/ml), *T. phagedenis* transformed with the shuttle plasmid pKMR in the presence of thrombin (2 µg/ml), or *T. phagedenis* transformed with *tp0751*/pKMR in both the presence (2 µg/ml) and absence of thrombin. Treponemes were grown anaerobically for 48 h at 37°C, and the size of the fibrin clots was analyzed visually at 0 and 48 h. Results are representative of six independent experiments. The yellow coloration present in the tubes of Panel C is due to TYGVS growth medium. (D) Fibrin clot degradation by *T. phagedenis* transformed with *tp0751*/pKMR in both the presence (2 µg/ml) and absence of thrombin was quantitated following 48 h growth at 37°C as described in (B). Student's two-tailed *t* test indicated that *T. phagedenis* transformed with *tp0751*/pKMR exhibited statistically significant levels of fibrinolysis compared to the levels exhibited by *T. phagedenis* transformed with pKMR alone (* indicates *p*<0.005).

Given the fact that pallilysin has been shown to degrade human fibrinogen [19], we predicted that it may also mediate degradation of insoluble fibrin, the major structural constituent of blood clots. To test this prediction, various forms of purified pallilysin were incubated with fibrin clots and the level of clot degradation was visually analyzed at selected time points. As shown in **Figure 8A**, wild-type pallilysin (C^24^-P^237^) that had not been pre-activated via removal of the pro-domain was capable of mediating complete degradation of fibrin clots between 24 and 48 h post-exposure. Fibrin clots incubated with autocatalytically activated wild-type pallilysin (T^93^-P^237^) showed more rapid degradation, with complete degradation observed between 0 and 24 h following exposure. In contrast, both water and the pallilysin active site mutant HEXXA (H^202^A) had no detectable effect on fibrin clot stability over the course of 48 h. To quantitate the fibrinolytic activity of recombinant pallilysin, the percent degradation of insoluble fibrin clots incubated with pallilysin over the course of 48 h was investigated. As shown in **Figure 8B**, wild-type pallilysin (C^24^-P^237^) and activated wild-type pallilysin (T^93^-P^237^) exhibited complete fibrin clot degradation by 48 h post-incubation, compared to minimal levels of clot degradation observed in the presence of water and the pallilysin active site mutant HEXXA (H^202^A) (*p*<0.0001).

To investigate the fibrinolytic potential of pallilysin within the context of a viable treponeme, an *in vitro* growth assay was performed to compare the capacity of *T. phagedenis* heterologously expressing pallilysin (*T. phagedenis_tp0751*/pKMR) to degrade fibrin clots with that of *T. phagedenis* transformed with the shuttle expression plasmid alone (*T. phagedenis*_pKMR). Treponemes were introduced to fibrin clots, grown until late exponential phase, and the level of clot degradation was visually analyzed at selected time points. As shown in **Figure 8C** (two right side panels), degradation of fibrin clots by *T. phagedenis_tp0751*/pKMR was clearly apparent following 48 h growth (late exponential phase; 1.25×10^9^±2.9×10^7^ cells ml^−1^ [mean ± standard error]). Fibrin clots incubated with *T. phagedenis_tp0751*/pKMR in the presence of thrombin (**Figure 8C**, second panel from right) exhibited increased levels of degradation compared to clots incubated with *T. phagedenis_tp0751*/pKMR in the absence of thrombin (**Figure 8C**, right panel). In contrast, when fibrin clots were incubated with *T. phagedenis*_pKMR in the presence of thrombin for 48 h (late exponential phase; 1.25×10^9^±5.0×10^7^ cells ml^−1^ [mean ± standard error]), minimal levels of fibrin clot instability were observed which were comparable to the levels observed when fibrin clots were incubated in treponemal growth medium alone (**Figure 8C**, two left side panels).

To quantitate the fibrinolytic activity of pallilysin-expressing *T. phagedenis*, the percent degradation of insoluble fibrin clots mediated by *T. phagedenis_*pKMR and *T. phagedenis_tp0751*/pKMR after 48 h growth was determined. As shown in **Figure 8D**, *T. phagedenis_tp0751*/pKMR grown in both the presence and absence of thrombin exhibited statistically significant levels of insoluble fibrin clot degradation (61%±5.8% and 54%±4.9% [mean ± standard error], respectively; *p*<0.005) when compared to the level of clot degradation observed in the presence of media alone or *T. phagedenis_*pKMR.

### Pallilysin is a target of opsonic antibodies

**Figure 9:**
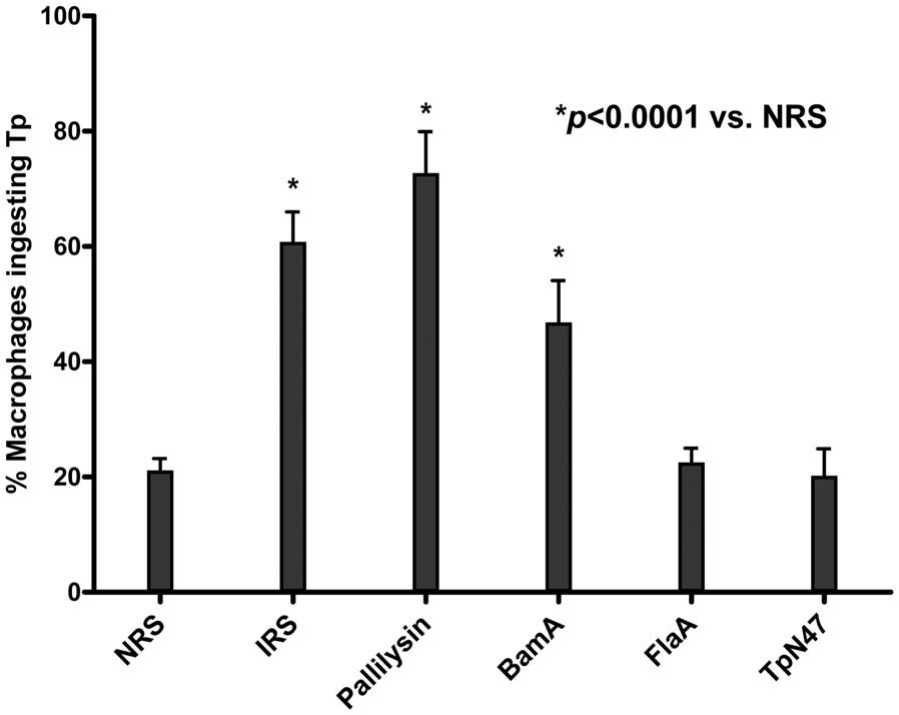
Pallilysin is a target of opsonic antibodies. *Treponema pallidum* was incubated with either normal rabbit serum (NRS), immune rabbit serum (IRS) (positive control), or polyclonal antiserum specific for pallilysin, BamA (positive control), FlaA (negative control), or TpN47 (negative control) and the percentages of rabbit macrophages phagocytosing *T. pallidum* (Tp) were calculated. Mean opsonization percentages from a minimum of 5 replicates are presented with bars indicating standard error (SE). For statistical analyses, opsonization of *T. pallidum* following exposure to each of the antisera was compared to *T. pallidum* opsonization after incubation with NRS using the Student's two-tailed *t* test. Exposure of *T. pallidum* to the two positive control antisera (BamA and IRS) and pallilysin-specific antiserum resulted in statistically significant levels of *T. pallidum* opsonization (*p*<0.0001) when compared to the level of *T. pallidum* opsonization observed following incubation with NRS (indicated by *).

In order to determine if pallilysin could be surface-exposed in *T. pallidum*, standard opsonophagocytosis assays were performed to determine the ability of pallilysin antiserum to opsonize *T. pallidum*. As shown in **Figure 9**, pallilysin-specific polyclonal antiserum exhibited significant opsonic activity compared with normal rabbit serum (*p*<0.0001). The level of opsonic activity observed for anti-pallilysin was similar to that of immune rabbit serum and consistently higher than the positive control BamA-specific antiserum (BamA/Tp0326; GenBank accession number, AAC65313, has been shown to be at least partially surface-exposed in *T. pallidum* [33], [34]). Importantly, the level of opsonic activity observed for antiserum generated against a periplasmic protein, FlaA (Tp0249; GenBank accession number, AAC65235), and a cytoplasmic membrane lipoprotein, TpN47 (Tp0574; GenBank accession number, AAC65545), was similar to the level of opsonic activity observed with normal rabbit serum (**Figure 9**), indicating the treponemal outer membranes remained intact throughout the course of the assays. These results suggest pallilysin is located on the surface of *T. pallidum*.

## Discussion

### Potential factors mediating *T. pallidum* invasion and dissemination

The highly invasive and disseminating nature of *T. pallidum* likely represents a multi-factorial process. Several mechanisms have been proposed to explain the invasiveness of *T. pallidum*, including treponemal adhesion [16], [19], [35], motility [36], [37], chemotaxis [15], unusual ultrastructure/low outer-membrane protein content [38], [39], host-inflammatory and immune responses [15], and antigenic variation [15], [40]. However, the factors mediating this striking pathogenic trait remain to be definitively identified. The current study further advances our understanding of *T. pallidum* pathogenesis by providing novel insight into the mechanism of pallilysin-mediated host component degradation, a pathogenic strategy we predict may be used by *T. pallidum* to facilitate bacterial dissemination and tissue invasion during the course of infection.

### Pallilysin is a host component-binding metalloprotease that coordinates zinc via an HEXXH motif

In the current study we further characterize the attachment and proteolytic capabilities of pallilysin, definitively establish that pallilysin is a zinc-binding metalloprotease, and demonstrate that the C-terminal HEXXH motif is located within the active site. ICP-MS analysis showed that H^198^, but not E^199^, is required for pallilysin zinc coordination, a finding that is consistent with the typical metalloprotease HEXXH motif whereby the glutamate functions as an essential general acid-base catalyst but is not directly involved in coordination of zinc [41], [42]. Additionally, the finding that mutation of the HEXXH active site residues abolishes host component proteolysis but does not adversely affect host component binding confirms the previously reported bifunctionality of pallilysin [19], in that the host component binding and proteolytic functionalities of pallilysin appear to be physically separate and functionally independent.

### Pro-pallilysin undergoes activation via inter-molecular autocatalysis

In the current study we demonstrate that each of the three pallilysin HEXXH active site mutants, which were purified under conditions identical to that of wild-type pallilysin, was stable following 24 h incubation at 37°C, whereas wild-type pallilysin exhibited characteristic self-cleavage. These results confirm that pallilysin, as opposed to contaminating proteases derived from the protein expression systems, solely mediates the observed post-purification pallilysin cleavage. Using Edman sequencing, we demonstrated that removal of the pallilysin N-terminal pro-domain occurs via autocatalytic cleavage between residues T^46^–A^47^ and Q^92^–T^93^. These results indicate that pallilysin's active site substrate subsites are able to accommodate very different amino acid types, which agrees with our evidence that the protease is capable of targeting multiple host proteins [19].

Using an approach similar to that used by Marie-Claire and colleagues [43], which directly tests the potential of a wild-type protease to process an active site mutant unable to undergo autocatalysis, the current study demonstrates that *in vitro* pallilysin maturation occurs via a sequential cleavage mechanism involving inter-molecular autocatalysis. It should be noted that the current *in vitro* study does not rule out the possibility of intra-molecular autocatalytic pallilysin activation occurring both *in vitro* and *in vivo*. Intra-molecular processing may be advantageous when pallilysin is present at very low concentrations. Our results also demonstrate that autocatalytically-cleaved pallilysin (T^93^-P^237^) exhibits enhanced rates of fibrinogenolysis compared to its full-length counterpart (C^24^-P^237^), indicating that it is in a more proteolytically active conformation.

### Pro-pallilysin is activated by thrombin cleavage

Eukaryotic convertases, such as the ubiquitously expressed furin, have been shown to activate bacterial toxins in the intracellular environment through limited proteolytic cleavage. Examples include the Shiga toxin, anthrax toxin, *Pseudomonas* exotoxin A, diphtheria toxin, botulinum toxin and tetanus toxin [44]–[46]. Furthermore, many bacterial proteases, such as the aspartic protease Pla from *Yersinia pestis*, the metalloprotease aureolysin from *Staphylococcus aureus*, and the LasB metalloprotease of *P. aeruginosa*, have been shown to cleave and activate host proteins, in particular components of the host plasminogen activation system [47]–[49]. To our knowledge the reverse mechanism, that is activation of bacterial proteases through limited cleavage by host proteases, has not been previously reported.

In the current study we have experimentally confirmed the presence of an N-terminal thrombin cleavage site between residues R^77^-S^78^ of pallilysin. Further, we have demonstrated that pallilysin truncated at the N-terminal thrombin cleavage site retains the ability to degrade human fibrinogen. The finding in the current study that non-His-tagged full-length pallilysin (C^24^-P^237^) exhibited enhanced fibrinogenolysis compared to truncated pallilysin (S^78^-P^237^) is consistent with the concept that pro-domains often function as intra-molecular chaperones essential for mediating correct protein folding and formation of the mature active protease [28], [50]. In light of this fact, it is possible that during recombinant expression truncated pallilysin (S^78^-P^237^) is incorrectly folded in the absence of this important molecular chaperone, which in turn results in sub-optimal proteolytic activity.

To our knowledge, this study represents the first published report describing a bacterial protease that may be activated via limited proteolysis mediated by host-expressed thrombin and thus may represent a novel paradigm in bacterial pathogenesis. The fact that *T. pallidum* encodes one of the smallest bacterial genomes identified to date [51] is consistent with the concept that the bacterium has evolved virulence mechanisms that exploit the biological function of host-encoded proteins for the promotion of pathogenicity during the course of infection. The functional redundancy of host-dependent (thrombin cleavage) and host-independent (autocatalysis) mechanisms of pallilysin activation may enable treponemal dissemination within the diverse host environments the bacterium encounters throughout the course of long-term infection. For example, thrombin levels within the skeletal and nervous systems, which *T. pallidum* is capable of infecting [15], [52], are significantly lower than levels found within the circulatory system [53].

### Pallilysin mediates degradation of fibrin clots

Localized coagulation is immediately induced in the host following inflammation and infection, functioning as one of the first lines of defense to limit tissue damage and promote healing [54]–[56]. Furthermore, recent studies have clearly demonstrated that fibrin clots are involved in the entrapment, localization, and killing of invasive strains of bacteria, including *Escherichia coli*, *Staphylococcus aureus*, and *Streptococcus pyogenes* [57], [58]. In the current study we demonstrate that purified recombinant pallilysin directly mediates fibrin clot dissolution. Furthermore, heterologous expression of pallilysin on the surface of *T. phagedenis* was shown to confer fibrinolytic activity upon this non-invasive model treponeme. Assuming this proteolytic capacity can be extended to *T. pallidum*, these results suggest this highly invasive pathogen may employ this rare bacterial fibrinolytic capability to inhibit and overcome the initial localized host-defense response raised against invading treponemes immediately following infection.

### The potential biological relevance of pallilysin in *T. pallidum* dissemination

The definitive identification of surface-exposed proteins in *T. pallidum* remains highly controversial, primarily due to the technical difficulties associated with working with *T. pallidum*. The current study employed opsonophagocytosis assays to demonstrate that pallilysin is a target of opsonic antibodies, which is consistent with localization of this protein to the outer leaflet of the outer membrane. Evidence that our laboratory has amassed from previous studies also strongly supports surface exposure of pallilysin in *T. pallidum*. Specifically, previous studies indicated that: (i) pallilysin is a lipoprotein that is exposed on the surface of our model treponeme, *T. phagedenis* [18], [19]; (ii) pallilysin-specific antibodies and pallilysin-specific peptides prevent attachment of viable *T. pallidum* to laminin-coated surfaces [17]; and (iii) viable, pallilysin-expressing *T. phagedenis* gains the ability to attach to host component-coated surfaces, an interaction that is inhibited by the presence of pallilysin-specific serum [18]. Furthermore, our contention that pallilysin is present on the surface of *T. pallidum* is consistent with the propensity for pallilysin to be cleaved by host-originating thrombin, to mediate host component binding and degradation, and by our finding that viable, pallilysin-expressing *T. phagedenis* gains the ability to degrade fibrin, as described herein. It has been shown that pallilysin is expressed during experimental and natural syphilis [16], [59]–[61], however, a prior transcriptome study that analyzed *T. pallidum* gene expression during experimental rabbit infection also indicated that pallilysin is transcribed at low-to-moderate levels during rabbit infection [62]. This suggests pallilysin's contribution to treponemal dissemination may result from localized host component degradation in the immediate vicinity of the invading treponemes, a concept supported by our results showing fibrin clot dissolution by pallilysin-expressing *T. phagedenis*.

**Figure 10:**
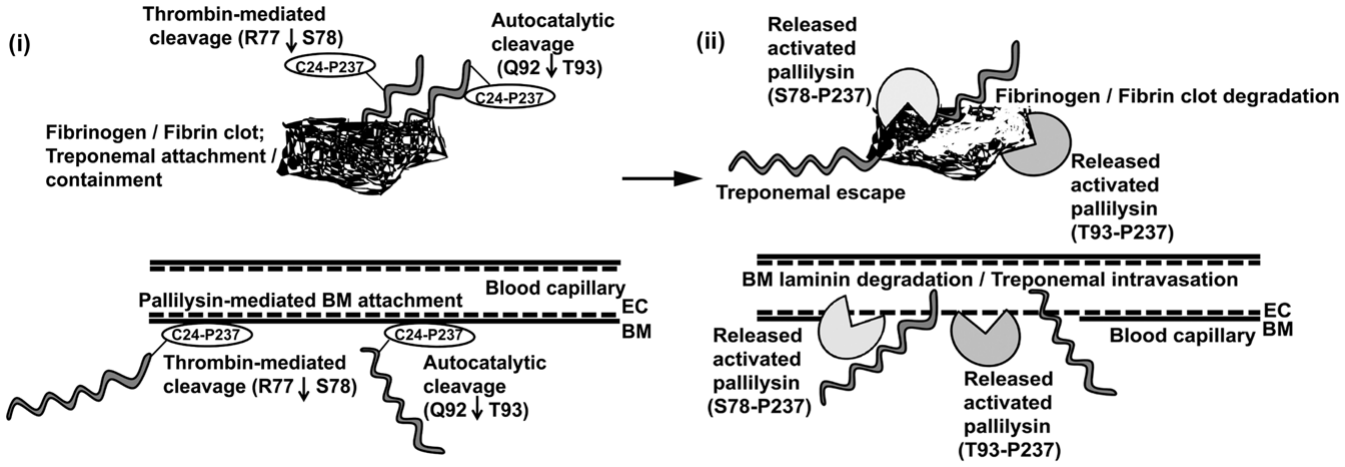
Model of the potential role of pallilysin in *T. pallidum* dissemination. (i) Surface-exposed pallilysin (C^24^-P^237^) mediates attachment of *T. pallidum* (<$>\scale 60%\raster="rg1"<$>) to host components, including the laminin-rich basement membrane (BM) that underlies the endothelial cells (EC) of blood vessels and to fibrinogen/fibrin clots. The N-terminal pro-domain of pallilysin is removed via host thrombin-mediated cleavage (cleavage at S^78^) or autocatalytic activation (cleavage at T^93^), which results in release of pallilysin from the treponemal surface into the external host milieu. (ii) Released pallilysin (S^78^-P^237^ and/or T^93^-P^237^) degrades host components located in the vicinity of the disseminating treponemes, including the laminin-rich BM and fibrin clots.

Collectively, the findings in the current study and previous studies [17]–[19] allow for the generation of a model to explain the potential role of pallilysin in *T. pallidum* dissemination and invasion (**Figure 10**). In this model, pallilysin would be produced as a lipoprotein and exported to the treponemal surface through a currently unknown mechanism. The adhesive functionality of pallilysin would facilitate attachment to host ECM components such as the laminin-rich basement membranes lining blood vessels and fibrinogen/fibrin clots. Previously, our laboratory identified specific pallilysin amino acid residues located between P^98^ and S^185^ that are critical for laminin-binding [17], suggesting that, at least for laminin, host component attachment is mediated via pallilysin residues C-terminal to the pallilysin thrombin and final autocatalytic cleavage sites. Therefore, cleavage of the pallilysin pro-domain is anticipated to result in release of the host component-bound activated protease from the treponemal surface. In this model, released pallilysin would promote localized host laminin and fibrinogen/fibrin degradation, which would facilitate basement membrane degradation/treponemal entry into the circulation and fibrin clot degradation, respectively, which would in turn promote bacterial dissemination and invasion.

## Materials and Methods

### Ethics statement

All animal studies were approved by the local institutional review boards at the University of Victoria and the University of Washington, and were conducted in strict accordance with standard accepted principles as set forth by the Canadian Council on Animal Care (CCAC), National Institutes of Health and the United States Department of Agriculture in facilities accredited by the American Association for the Accreditation of Laboratory Animal Care and the CCAC.

### Bacteria

*T. pallidum* subsp. *pallidum* (Nichols strain) was propagated in, and extracted from, New Zealand White rabbits as described elsewhere [63]. *T. phagedenis* biotype Kazan, which had been previously transformed with either the shuttle plasmid pKMR4PEMCS (pKMR) or the full length pallilysin-expressing construct *tp0751*/pKMR4PEMCS (*tp0751*/pKMR [18]), were grown in an atmosphere of 97.5% N_2_ and 2.5% H_2_ in a Coy Laboratory Products anaerobic chamber (Mandel Scientific Company Inc., Guelph, ON) at 37°C in TYGVS medium [64] supplemented with 20% heat-inactivated rabbit serum (Sigma) in the presence of 10 µg/ml rifampicin and 40 µg/ml erythromycin.

### Host proteins

Plasminogen-depleted human fibrinogen (Calbiochem) was purchased from VWR International (Mississauga, ON). Thrombin isolated from human plasma and laminin isolated from Engelbreth-Holm-Swarm murine sarcoma basement membrane were purchased from Sigma-Aldrich Canada Ltd. (Oakville, ON).

### Construct cloning and site-directed mutagenesis

Pallilysin (*tp0751*) DNA fragments encoding amino acid residues C^24^-P^237^ and S^78^-P^237^ and *tp0453* DNA encoding amino acid residues A^32^-S^287^ were PCR amplified from *T. pallidum* subsp. *pallidum* (Nichols strain) genomic DNA using the primers listed in **Table S1**. Pallilysin amplicons were cloned into the T7 promoter destination expression vectors, pDEST-14 and pDEST-17 (Gateway technology, Invitrogen), according to the manufacturer's instructions. The pDEST-14 and pDEST-17 expression vectors allow for the generation of tagless and N-terminally fused hexahistidine-tagged recombinant proteins, respectively. The negative control, Tp0453, was cloned as previously described [19]. For site-directed mutagenesis of the pallilysin HEXXH motif, the pDEST-17 pallilysin (C^24^-P^237^) construct described above was subjected to three independent amino acid substitutions, AEXXH (H^198^A), HAXXH (E^199^A), and HEXXA (H^202^A) using the primers listed in **Table S1** and the QuikChange site-directed mutagenesis kit (Stratagene; purchased from VWR International, Mississauga, ON), according to the manufacturer's instructions. All constructs were confirmed as correct by DNA sequencing.

### Recombinant protein expression and purification

In order to ensure consistent levels of protein purity between wild-type and mutant forms of pallilysin, all recombinant constructs (wild-type and mutant) were expressed and purified under identical conditions, as previously described [19], with the following modifications. FPLC purification of soluble tagless and histidine-tagged recombinant proteins was performed using immobilized metal ion affinity chromatography. Following loading of the recombinant proteins onto 1 ml HisTrap FF affinity columns (GE healthcare, Baie D'Urfe, QC) pre-packed with precharged nickel sepharose 6 fast flow resin, the columns were washed with buffer containing zinc chloride and calcium chloride at pH 7.0 (20 mM HEPES pH 7.0, 500 mM sodium chloride, 20 mM imidazole, 10 µM zinc chloride, 25 mM calcium chloride, 1% glycerol). Bound recombinant proteins were eluted with a zinc/calcium-containing elution buffer (20 mM HEPES pH 7.0, 500 mM sodium chloride, 250 mM imidazole, 10 µM zinc chloride, 25 mM calcium chloride, 1% glycerol). In order to further ensure that all proteins were free of potential contaminants originating from the *E. coli* expression strain, concentrated recombinant proteins were loaded onto, and eluted from, a gel filtration column (HiLoad 16/60 Superdex 75; GE Healthcare) using calcium-containing gel filtration buffer (20 mM HEPES pH 7.0, 150 mM sodium chloride, 25 mM calcium chloride, and 1% glycerol). Identical gel filtration fraction ranges were collected from each of the wild-type and mutant pallilysin purifications. All recombinant proteins were deemed to be purified to homogeneity as indicated by SDS-PAGE analysis (**Figure 1**), mass spectrometry and Edman degradation (as described below).

### Normal rabbit serum, immune rabbit serum and polyclonal antibody production

The following recombinant proteins were purified and subsequently used for rabbit immunizations: Pallilysin (amino acids 54–173) [17], BamA (amino acids 25–837) [33], TpN47 (amino acids 34–459) [65], FlaA (amino acids 33–416) [65], and Tp0453 (amino acids 30–287) [61]. Polyclonal antiserum specific for pallilysin (Tp0751), BamA (Tp0326), FlaA (flagellar protein, Tp0249), TpN47 (cytoplasmic membrane lipoprotein, Tp0574), and Tp0453 were raised in New Zealand rabbits as previously described [17], [33]. Normal rabbit serum (NRS) was collected prior to the initial immunizations. Immune rabbit serum (IRS) was collected from rabbits infected with *T. pallidum* for >90 days.

### Inductively coupled plasma-mass spectrometry

Zinc ion content in wild-type and mutant pallilysin was analyzed at Cantest Ltd. (Burnaby, BC) using Inductively Coupled Plasma-Mass Spectrometry (ICP-MS). Briefly, wild-type pallilysin, the active site mutants (AEXXH [H^198^A] and HAXXH [E^199^A]), as well as gel filtration buffer (20 mM HEPES pH 7.0, 150 mM sodium chloride, 25 mM calcium chloride, and 1% glycerol) were dispensed into individual 15 ml polypropylene tubes. The four samples were each reacted with 200 µl of nitric acid, and heated in a 95°C water bath for 1 h. The digested solutions were made up to a final volume of 5 ml with de-ionized water. The samples were analyzed by conventional ICP-MS at a dilution of 5x. The metal element Zn was fully quantified against a certified standard using single point calibration. Sample analysis and operation of the ICP-MS was done according to Cantest's in-house standard operating procedures.

### ELISA-based binding assays

To test for adherence of recombinant wild-type pallilysin (C^24^-P^237^), pallilysin active site mutants (AEXXH [H^198^A], HAXXH [E^199^A], and HEXXA [H^202^A]), and Tp0453 (negative control) to fibrinogen and laminin, ELISA-based assays were performed as previously described [16] with the following modifications. All ELISA-based assays were performed using Greiner plates (VWR International). Recombinant proteins were diluted in TBS (Tris-Buffered Saline) and wells were washed with TBS-0.1% Tween 20 (TBS-T). Primary antibodies were pre-absorbed overnight in 5% skim milk powder in TBS-T using an *E. coli* lysate expressing an irrelevant His-tagged recombinant protein. Adherent recombinant proteins were detected using a 1∶2,500 dilution of anti-pallilysin or anti-Tp0453 serum (diluted in TBS-T). The secondary antibody (goat anti-rabbit IgG conjugated to HRP [Sigma-Aldrich Canada Ltd.]) was used at a 1∶10,000 dilution. Wells were developed using the *o*-Phenylenediamine dihydrochloride (OPD) substrate system (Sigma-Aldrich Canada Ltd.) and the reaction was stopped with a 1∶10 dilution of H_2_S0_4._ Plates were read at 492 nm with a Spectra Max 5 ELISA plate reader (Molecular Devices, Sunnydale, CA). Statistical analyses were performed using the Student's two-tailed *t* test.

### Fibrinogen degradation assays

Fibrinogen degradation assays were performed as previously described [19] with the following modifications. *In vitro* fibrinogen degradation assays (SDS-PAGE-based) were performed using recombinant pallilysin (C^24^-P^237^) (tagless and His-tagged wild-type and His-tagged mutants), pallilysin (S^78^-P^237^) (tagless wild-type), pallilysin pre-incubated at 37°C for 22 h (T^93^-P^237^) (His-tagged), and the negative control, Tp0453 (His-tagged). Recombinant proteins were incubated with plasminogen-free human fibrinogen in a HEPES-based protease activation buffer at pH 7.0 (20 mM HEPES, pH 7.0, 25 mM CaCl_2_). For the fibrinogen degradation fluorescence resonance energy transfer (FRET) assays, recombinant proteins were diluted in HEPES-based activation buffer at pH 7.0 (20 mM HEPES pH 7.0, 25 mM CaCl_2_) and added in replicates of between four and eight to sterile Corning Costar 96-well plates (Fisher Scientific). Blank sample wells consisted of HEPES activation buffer in the absence of recombinant proteins.

### *In silico* analysis

Pallilysin was analysed for the presence of predicted protease cleavage sites using the peptide cutter program [31] (http://web.expasy.org/peptide_cutter/) and manually analysed for the presence of potential thrombin cleavage sites by comparison to the MEROPS [32] thrombin cleavage site specificity matrix (http://merops.sanger.ac.uk/cgi-bin/pepsum?id=S01.217type=P). MEROPS is a freely available online database for proteases and protease inhibitors developed at the Wellcome Trust Sanger Institute.

### Pallilysin autocatalysis, thrombin cleavage and N-terminal sequencing

Pallilysin autocatalytic activity was monitored by incubating recombinant wild-type (500 µg) and pallilysin HEXXH active site mutants (500 µg) at 37°C for 24 h. Protein samples (10 µg) were removed hourly throughout the 24 h incubation, mixed with 10 µl of sample buffer (20% glycerol [w/v], 0.125 M Tris-HCl pH 6.8, 10% β-mercaptoethanol [v/v], 8% SDS [w/v], and 0.02% bromophenol blue [w/v]), heated at 95°C for 10 min and protein self-lysis analysed by electrophoresis in 15% polyacrylamide gels at a constant voltage of 200 Volts for 1 h. Gels were stained in Coomassie Brilliant Blue R-250 solution (0.25% [w/v] Coomassie Brilliant Blue R-250, 7.0% [v/v] acetic acid, and 40.0% [v/v] methanol) and destained in 5% (v/v) acetic acid, 20% (v/v) methanol, 75% (v/v) water.

To determine whether pallilysin undergoes intra- or inter-molecular autocatalysis, wild-type full-length pallilysin (C^24^–P^237^) (100 µg) was incubated with an equal amount of pallilysin active site mutant (AEXXH [H^198^A]) at 37°C for 22 h. Protein samples (10 µg) were removed at 0 and 22 h post-incubation and analyzed for autocatalytic processing as described above. Autocatalytic cleavage products were compared to the autocatalytic cleavage products generated from wild-type pallilysin and AEXXH (H^198^A) mutant pallilysin incubations.

Cleavage of recombinant proteins with thrombin was performed using the Thrombin CleanCleave kit (Sigma) according to the manufacturer's instructions. Briefly, 100 µg of wild-type pallilysin (C^24^-P^237^) and the negative control, Tp0453, was incubated at 20°C for 24 h with 30 µl of thrombin-agarose resin (4.5 µg of thrombin) in the presence of 10 mM calcium chloride. Protein samples (10 µg) were removed at 0, 1, 2, 4, 6, and 24 h post-incubation and analyzed for thrombin cleavage using SDS-PAGE as detailed above.

For Edman sequencing, autocatalytic peptides (20 µg total protein loaded per lane) and thrombin-cleavage fragments (10 µg total protein loaded per lane) were electrophoresed on 16% and 12% precast Novex Tris-glycine gels (Invitrogen), respectively. Proteins were transferred to Millipore Immobilon-P polyvinylidene fluoride (PVDF) membranes (Fisher scientific) at 200 mA for 1 h. Membranes were washed extensively in ultrapure water to remove SDS and glycine and stained in 0.1% Coomassie Brilliant Blue R250 solution (0.1% Coomassie Brilliant Blue R250, 40% methanol, 1% acetic acid, 58.9% ultrapure water) for 60 seconds. Membranes were destained in ultrapure water for 1 h at 20°C, followed by a 15 min destain in 50% methanol and 3×10 min destain steps in ultrapure water. Membranes were air-dried for 16 h and pallilysin autocatalytic protein bands and the thrombin-cleavage peptide corresponding to approximately 18 kDa were excised from the PVDF membranes. N-terminal sequence analysis of excised protein bands was performed using Edman chemistry on an Applied Biosystems (AB) Procise liquid-pulse protein sequenator at the Protein and Nucleic Acid (PAN) facility, Stanford University (CA). Briefly, PTH (phenylthiohydantoin)-amino acids were separated on a Brownlee C-18 reverse phase column (2.1 mm×22 cm) at 55°C using a linear gradient of Buffer A (3.5% tetrahydrofuran and 2% proprietary AB premix buffer concentrate [approximately 10–20% acetic acid, <10% sodium acetate, <10% sodium hexanesulfonate, 65–75% water]) and 14–44% buffer B (12% isopropanol in acetonitrile) over 17.6 min. PTH-amino acid analysis was performed using model 610A software, version 2.1.

### Fibrin clot degradation assays

In order to determine if recombinant pallilysin is capable of degrading insoluble fibrin clots, 60 µl plasminogen-free human fibrinogen (5.0 mg/ml) was incubated with 50 µl human thrombin (5 U/ml in 50 mM sodium chloride, 0.1% bovine serum albumin [BSA], 5 mM calcium chloride) for 30 min at 37°C. Excess buffer was removed, the resulting clot weighed (time 0 h), and then the clots were incubated with water (negative control) or 40 µg of each recombinant protein (pallilysin HEXXA [H^202^A] active site mutant, wild-type pallilysin [C^24^-P^237^] and autocatalytically activated wild-type pallilysin [T^93^-P^237^]) for 48 h at 37°C. At 0, 24, and 48 h post-incubation digital pictures were taken to record fibrin clot size. The assay was repeated seven times.

To determine if pallilysin heterologously expressed in the culturable spirochete *T. phagedenis* is capable of mediating fibrin clot degradation, *T. phagedenis* transformed with the pallilysin expression construct *tp0751*/pKMR was compared with *T. phagedenis* transformed with the shuttle vector alone (pKMR) for the ability to degrade fibrin clots. Transformation of *T. phagedenis* and demonstration of pallilysin surface expression was previously performed by Cameron and co-workers [18]. Fibrin clots were prepared as described above and incubated anaerobically for 48 h with either TYGVS medium alone, *T. phagedenis* transformed with pKMR, or *T. phagedenis* transformed with *tp0751*/pKMR. When present, human thrombin (Sigma) was added to a final concentration of 2 µg/ml. At 0 h (lag phase; 2.4×10^7^±7.6×10^5^ cells ml^−1^ [mean ± standard error]) and 48 h (late exponential phase; 1.25×10^9^±2.2×10^7^ cells ml^−1^ [mean ± standard error]) post-incubation, digital pictures were taken to record fibrin clot size. Darkfield microscopy with a Nikon Eclipse E600 microscope (Nikon Canada, Mississauga, ON) was used for cell counts and to ensure treponemes remained viable and motile throughout the experiment. The assay was repeated six times.

A decrease in clot weight following the 48 h incubations (expressed as percent decrease) was used to quantitate the level of clot degradation. Following 48 h incubation, all buffers, recombinant proteins or cultures were carefully removed from the fibrin clots by pipette aspiration and low speed centrifugation (3 seconds at 100× *g*) and clot weight was measured and compared with the clot weight at time 0 h. All measurements were performed using a Denver instrument S-114 analytical balance (Fisher Scientific, Ottawa, ON). Statistical analyses were performed using the Student two-tailed *t* test. Clot quantitation assays were repeated three times.

### Opsonophagocytosis assays

NRS, IRS, and polyclonal antisera generated against recombinant pallilysin, BamA (Tp0326; positive control), TpN47 (Tp0574; negative control), and FlaA (Tp0249; negative control), as described above, were tested for their ability to opsonize *T. pallidum* using standard opsonophagocytosis assays as previously described [33], [66]. Opsonophagocytosis assays were repeated at least twice.

### GenBank accession numbers

**Tp0249 (FlaA)**; AAC65235, **Tp0326 (BamA)**; AAC65313, **Tp0453**; AAC65443, **Tp0574 (TpN47)**; AAC65545, **Tp0751 (Pallilysin)**; AAC65720

## Supporting Information

Table S1 **Primers used to amplify DNA for recombinant protein expression.**
(DOC)
