# Supplementary material for: Activation and Proteolytic Activity of the Treponema pallidum Metalloprotease, Pallilysin
Source: PLoS Pathog. 2012 Jul 26;8(7):e1002822. doi: 10.1371/journal.ppat.1002822 (PMC3406077; doi:10.1371/journal.ppat.1002822)
Supplement: Table S1 — Primers used to amplify DNA for recombinant protein expression. (DOC) [file ppat.1002822.s001.doc]

| **ORF** | **Primer Orientation** | **Sequence^1^** |
| --- | --- | --- |
| Wt Pallilysin (C^24^-P^237^) | Sense | 5´ -GGGGACAAGTTTGTACAAAAAAGCAGGCTGCTTTCAGCACGGTCAC |
|  | Antisense | 5´ -GGGGACCACTTTGTACAAGAAAGCTGGGTTTCAGGGCGAAGGAGCACTAG |
| Pallilysin (S^78^-P^237^ tagless) | Sense | 5´ -CACCATGTCACATGGAAACGCCCCG |
|  | Antisense | 5´ -GGGGACAAGTTTGCACGGTCACGTTC |
| Pallilysin (C^24^-P^237^ tagless) | Sense | 5´ -CACCATGTGCTTTCAGCACGGTCACG |
|  | Antisense | 5´ -GGGGACAAGTTTGCACGGTCACGTTC |
| Pallilysin AEXXH (H^198^A) | Sense | 5´ -GGTAACATTTGAATCCGCCGAGGTGATACACGTAAGGG |
|  | Antisense | 5´ -CCCTTACGTGTATCACCTCGGCGGATTCAAATGTTACC |
| Pallilysin HAXXH (E^199^A) | Sense | 5´ -GGTAACATTTGAATCCCACGCGGTGATACACGTAAGGG |
|  | Antisense | 5´ -CCCTTACGTGTATCACCGCGTGGGATTCAAATGTTACC |
| Pallilysin HEXXA (H^202^A) | Sense | 5´ -CCCACGAGGTGATAGCCGTAAGGGCGGTTGAAG |
|  | Antisense | 5´ -CTTCAACCGCCCTTACGGCTATCACCTCGTGGG |
| Tp0453 | Sense | 5´ -CTAGAC**CATATG**GCATCAGTAGATCCGTTGG |
|  | Antisense | 5´ -GTCAG**CTCGAG**TCACGAACTTCCCTTTTTGGAG |

^1^ Restriction sites are highlighted in bold. Base changes resulting in the site-directed mutations are underlined.
